# Anthrax Toxin Receptor 2 Determinants that Dictate the pH Threshold of Toxin Pore Formation

**DOI:** 10.1371/journal.pone.0000329

**Published:** 2007-03-28

**Authors:** Heather M. Scobie, John M. Marlett, G. Jonah A. Rainey, D. Borden Lacy, R. John Collier, John A.T. Young

**Affiliations:** 1 Infectious Disease Laboratory, The Salk Institute for Biological Studies, La Jolla, California, United States of America; 2 Cellular and Molecular Biology Graduate Program, University of Wisconsin-Madison, Madison, Wisconsin, United States of America; 3 Department of Microbiology and Immunology, Vanderbilt University Medical Center, Nashville, Tennessee, United States of America; 4 Department of Microbiology and Molecular Genetics, Harvard Medical School, Boston, Massachusetts, United States of America; Baylor College of Medicine, United States of America

## Abstract

The anthrax toxin receptors, ANTXR1 and ANTXR2, act as molecular clamps to prevent the protective antigen (PA) toxin subunit from forming pores until exposure to low pH. PA forms pores at pH ∼6.0 or below when it is bound to ANTXR1, but only at pH ∼5.0 or below when it is bound to ANTXR2. Here, structure-based mutagenesis was used to identify non-conserved ANTXR2 residues responsible for this striking 1.0 pH unit difference in pH threshold. Residues conserved between ANTXR2 and ANTXR1 that influence the ANTXR2-associated pH threshold of pore formation were also identified. All of these residues contact either PA domain 2 or the neighboring edge of PA domain 4. These results provide genetic evidence for receptor release of these regions of PA as being necessary for the protein rearrangements that accompany anthrax toxin pore formation.

## Introduction

Anthrax toxin is a major virulence factor of *Bacillus anthracis* that is thought to cause many of the symptoms observed in anthrax disease. The toxin consists of a single receptor-binding subunit, protective antigen (PA), and two catalytic subunits: lethal factor (LF), a metalloprotease that cleaves and inactivates MKKs [1]–[3], and edema factor (EF), a calmodulin-dependent adenylate cyclase that converts ATP into cAMP [4], [5]. PA binds to either of two host cell receptors, ANTXR1 (anthrax toxin receptor, ATR/TEM8) and ANTXR2 (capillary morphogenesis gene 2, CMG2) [6], [7]. After binding the cellular receptor, the 83 kD form of PA, (PA_83_), is cleaved by cell-surface furin into a 63 kD form, (PA_63_), which goes on to heptamerize into a ring, or pre-pore [8]. Alternatively, cell surface receptors may engage the cleaved PA_63_, which exists as monomers or multimers in the blood of infected animals [9], [10]. The toxin-receptor complex is internalized by a mechanism involving LRP6 [11], and trafficked to a low pH endocytic compartment where acid pH triggers pore formation and translocation of the catalytic moieties into the cytoplasm [12]–[14].

PA is comprised of four protein domains with different functions: following PA_20_ removal and PA_63_ oligomerization, domain 1 binds EF and/or LF; domain 2 is involved in pore formation, EF/LF translocation, PA_63_ oligomerization, and receptor binding; domain 3 in PA_63_ oligomerization; and domain 4 in receptor binding [15]. PA binds to a von Willebrand factor A (vWA) domain that is common to both receptors and most similar to the inserted (I) domains of α-integrins [16]. Similar to α-integrin-ligand interactions, a carboxylate side-chain from PA domain 4 (residue D683) directly coordinates the divalent cation bound at the receptor metal ion dependent adhesion site (MIDAS) [17], [18]. The co-crystal structure of the ANTXR2 I domain bound to PA has shown that the contact surface between these two proteins is much larger (∼2000 Å^2^) than typical α-integrin-ligand binding (∼1300 Å^2^) [17], [18]. The extensive contact of ANTXR2 with PA domains 2 and 4 most likely accounts for the very high affinity of the ANTXR2 I domain-PA interaction (K_D_ = 170 pM) [19]. By contrast, the ANTXR1 I domain-PA interaction exhibits a much lower binding affinity (K_D_ = 130 nM) [20].

In the absence of receptor, PA_63_ forms pores at neutral pH, while low pH is required for pore formation when the toxin subunit is bound to receptor. Thus it has been proposed that each receptor acts as a molecular clamp to restrict pore formation until the complex encounters an acidic endosomal compartment where low pH induces structural changes in the PA heptameric pre-pore leading to pore formation. In this model, receptor contact with the base (residues 340–348) of the PA domain 2 membrane insertion loop (β2- β3; residues 285–340) restrains pore formation until protonation of PA and/or ANTXR2 residues loosens this interaction to allow domain 2 to undergo a large conformational change and form an extended β-barrel pore [17], [18], [21], [22]. The pH required for pore formation when PA is bound to ANTXR2 (pH ∼5.0) is fully one unit lower than when it is bound to ANTXR1 (pH ∼6.0), suggesting that the receptor might dictate the subcellular location of pore formation [14], [23]. Based upon the difference in pH threshold for pore formation, ANTXR1 is considered to be the weaker molecular clamp, being released more easily from PA than is ANTXR2. Consistent with these pH requirements, ANTXR2-, but not ANTXR1-, mediated cellular intoxication is blocked by ammonium chloride (NH_4_Cl) treatment, which raises endosomal pH [14].

Prior to this report it was not known which receptor determinants are responsible for dictating the receptor-specific pH thresholds of anthrax toxin pore formation. Here we identify these determinants of ANTXR2 and show that they are involved with binding PA domain 2 and the neighboring edge of PA domain 4.

## Methods

### DNA constructs, cell transfections and protein production

QuikChange mutagenesis (Stratagene) was performed on ANTXR2-EGFP (CMG2^489^-EGFP) [7] or ANTXR1-EGFP (ATR/TEM8 sv2-EGFP) [6] plasmids with oligonucleotide primers described in Table S1. For transient receptor expression, ∼5×10^6^ CHO-R1.1 cells were transfected with 1 or 3 µg plasmid DNA ANTXR1- or ANTXR2-EGFP and 5 or 3 µg pBSII KS(-) carrier DNA using Lipofectamine2000 (Invitrogen). All constructs were confirmed by DNA sequencing. WT PA protein was isolated from the periplasm of *E. coli* [24], and purified as previously described [25]. LF_N_-DTA (the N-terminal, PA-binding domain of LF fused to the catalytic A chain of diphtheria toxin, which kills cells when internalized) was produced as previously described [26].

### Cell intoxication assays

At 24 hours post-transfection, triplicate samples of ∼7×10^5 ^cells were plated in 12-well culture dishes and pre-incubated for 1 hour with 30 mM NH_4_Cl, or media only. Then cells were incubated for 6 hours with 5×10^−8^ M PA and 10^−10^ M LF_N_-DTA, or LF_N_-DTA alone, at which point the media was changed and all samples were incubated with 30 mM ammonium chloride changed every 6 hours for ∼18 hours. Cells were analyzed by flow cytometry and the percentage of live, EGFP-positive cells in the presence of PA and LF_N_-DTA was divided by that in the LF_N_-DTA alone sample (no toxin killing) to determine the relative EGFP cell viability. WT ANXTR2-expressing cells are normally susceptible to an NH_4_Cl block to intoxication, while ANTXR1-expressing cells are resistant [14]. Cytosolic EGFP-expressing cells were used as a negative control for intoxication (data not shown).

### PA pore formation on cell surfaces

At 48 hours post-transfection, ∼2×10^6^ cells were pre-incubated in supplemented F12 media with dGAB cocktail [50 mM 2-deoxy-glucose (Sigma), 10 mM sodium azide (Sigma), 200 nM bafilomycin A1 (Alexis Biochemicals)] for 45 minutes at 37°C. Cells were then bound with 10^−8^ M PA_63_ (List Labs) in the presence of dGAB for 2 hours at 4°C. Pore formation was induced by incubation for 10 minutes at 37°C with PBS buffered with Tris pH 6.8, Tris pH 6.5, MES pH 6.0, MES pH 5.6, MES pH 5.4 and Sodium Acetate pH 5.0 or alternatively MES pH 6.0, MES pH 5.8, MES pH 5.6, MES pH 5.4, Sodium Acetate pH 5.2, and Sodium Acetate pH 5.0 respectively (final pH). Samples were analyzed as previously described [14].

## Results

### Homolog-scanning mutagenesis of ANTXR2

**Figure 1:**
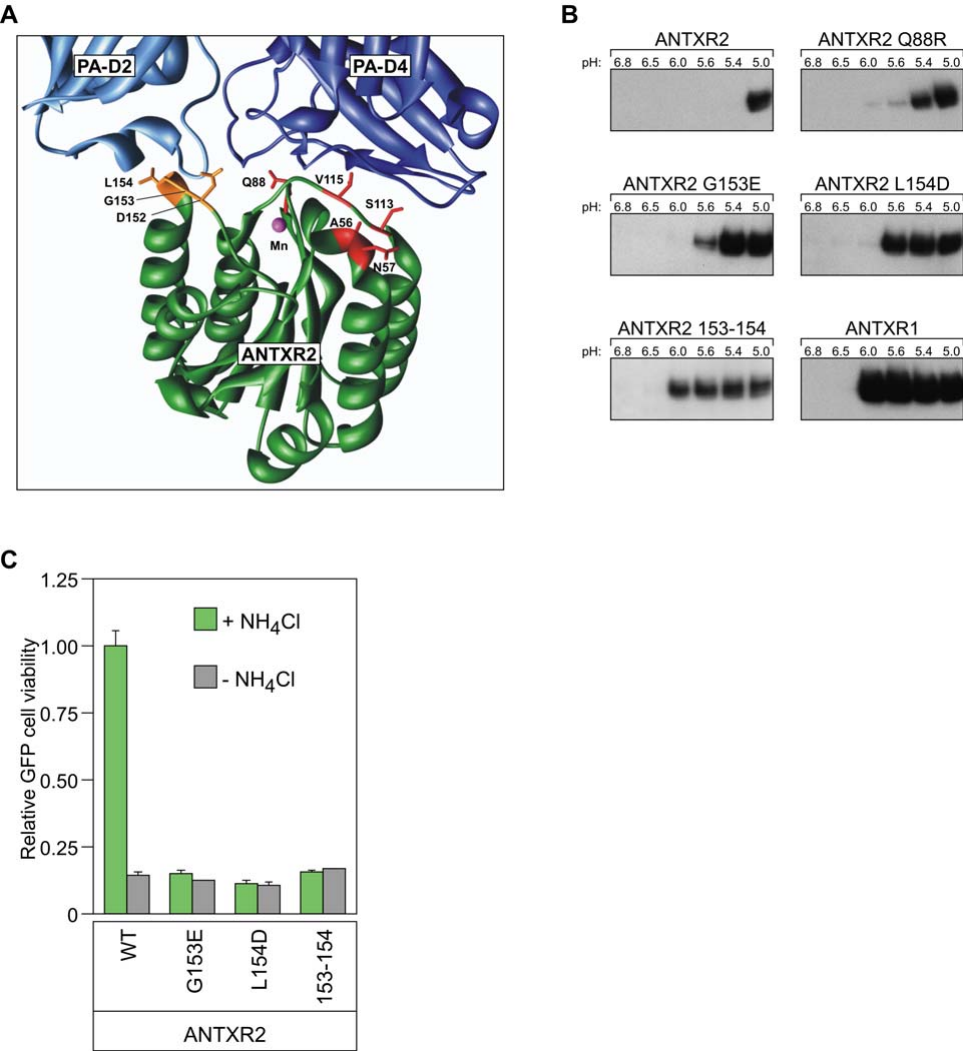
Mutagenesis of ANTXR2. (A) Ribbon model of ANTXR2-PA binding interface (UCSF Chimera; PDB# IT6B) [17]. PA domain 2 (PA-D2) and PA domain 4 (PA-D4) are colored light and dark blue, respectively, and the ANTXR2 I domain is colored dark green. The Mn^2+^ cation bound by the ANTXR2 MIDAS is shown in magenta. ANTXR2 residues that are not conserved in ANTXR1 and are involved in PA-D2 and PA-D4 contacts are depicted in orange and red stick representation, respectively. (B) Cells transiently expressing ANTXR2-EGFP or mutant ANTXR2-EGFP receptors treated with dGAB for 45 mins at 37°C were incubated with PA_63_ at 4°C in the presence of dGAB to prevent PA internalization, and pore formation was induced by exposure to low pH buffers before lysing cells. PA pores, which correspond an SDS-resistant high molecular weight species, were detected by SDS-PAGE and immunoblotting with an anti-PA serum followed by an HRP-conjugated secondary antibody. The high molecular weight, SDS-resistant species on the blot representing PA pore are shown. These data are representative examples of at least three similarly performed independent experiments. (C) Triplicate samples of cells expressing WT ANTXR2-EGFP, and ANTXR2-EGFP with G153E, L154D, and 153–154 mutations were treated with PA and LF_N_-DTA in the presence or absence of 30 mM NH_4_Cl and assayed for cell viability as described in materials and methods.

We reasoned that non-conserved amino acid residues located at the PA-binding interface were likely to be responsible, at least in part, for the distinct receptor-specific pH thresholds of anthrax toxin pore formation. There are eight such residues in ANTXR2 (A56, N57, Q88, S113, V115, D152, G153, and L154), which correspond to Leu, His, Arg, Leu, Gly, His, Glu, and Asp, respectively, in ANTXR1 [17], [18] (Fig. 1A). To test their involvement, each ANTXR2 residue was independently replaced by the corresponding ANTXR1 residue in the context of an ANTXR2-EGFP fusion protein [7]. The altered receptors were expressed in transiently-transfected CHO-R1.1 cells, which lack PA receptors [6]. The pH threshold of PA pore formation was then measured in each case using a previously described assay [14] (Fig. 1B).

Among the mutations in PA domain 2 contact residues, the D152H mutation had almost no effect on the pH threshold of pore formation (data not shown). By contrast, the G153E and L154D amino acid substitutions weakened the ANTXR2 molecular clamp, allowing PA pore formation at a pH value that was 0.6 units higher than that seen with wild-type ANTXR2 (Fig. 1B). Indeed, when these two mutations were combined (construct ANTXR2 153–154), ANTXR2 was fully converted into a receptor with the pore-inducing properties of ANTXR1 (Fig. 1B). Consistently, the G153E, L154D and 153–154 amino acid substitutions rendered cellular intoxication via ANTXR2, which is normally sensitive to inhibition by NH_4_Cl treatment, resistant to that treatment (Fig. 1C).

Among the mutations tested in PA-domain 4 contact residues all had almost no effect on the pH of pore formation (data not shown) with the exception of the Q88R mutation that weakened the molecular clamp, allowing PA pore formation at pH 5.4 (Fig. 1B). These data indicated that residues G153 and L154, located in the β4-α4 loop of the receptor I domain, which contacts PA domain 2, and residue Q88 which contacts PA domain 4, are major determinants of the lower pH threshold requirement associated with ANTXR2.

### Homolog-scanning mutagenesis of ANTXR1

**Figure 2:**
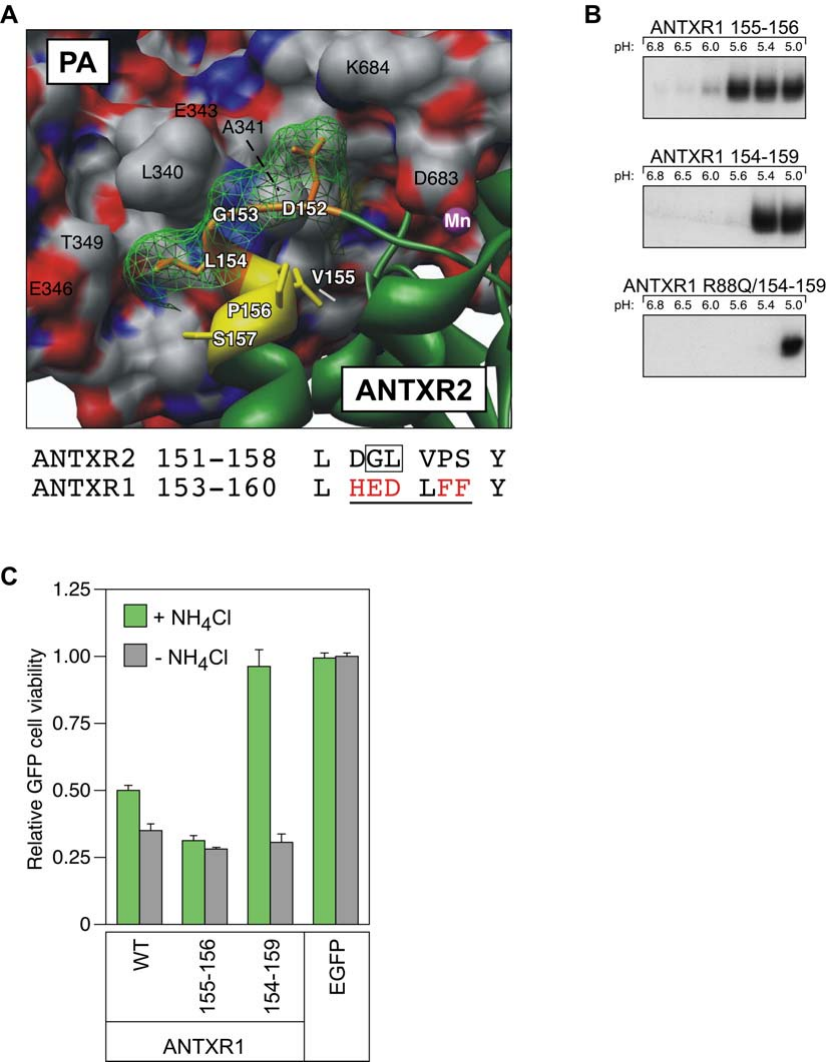
Mutagenesis of ANTXR1. (A) Structural diagram of the unique ANTXR2 residues involved in PA domain 2 contact. Residues 152–154 are depicted in orange stick representation with a mesh space-fill overlay, and those residues (155–157) that may affect display of the upstream contact residues are depicted in yellow sticks. The numbered residues of ANTXR2 are shown in white type. PA is depicted in space-fill representation, where C is grey, N is blue and O is red and the numbered residues are shown in black type. Below is an alignment of the primary sequence for this region in ANTXR2 and ANTXR1. The residues depicted in orange and yellow above are underlined; non-conserved changes in ANTXR1 are highlighted in red; and the ANTXR2 G153 and L154 residues shown to be important in Fig. 1 are boxed. (B) PA pore formation was assayed at different pH values on the surfaces of cells transiently expressing ANTXR1-EGFP and mutant ANTXR1-EGFP receptors, as described in Fig. 1B. These data are representative examples of at least three similarly performed independent experiments. (C) Triplicate samples of cells were assayed for intoxication with PA and LF_N_-DTA in the presence or absence of 30 mM NH_4_Cl and analyzed for cell viability as described in Fig. 1C.

To determine their sufficiency for the low pH threshold of pore formation associated with ANTXR2, residues G153, L154, and Q88 were introduced at the corresponding positions of ANTXR1. The ANTXR1 155–156 protein, with ANTXR2 residues G153 and L154 replacing the corresponding residues E155 and D156 of ANTXR1, displayed only a modest (0.4 pH unit) shift in threshold of PA pore formation (Fig. 2A and Fig. 2B). Consistently, intoxication via this mutant receptor was still inhibitable by NH_4_Cl (Fig. 2C). Therefore, these two residues conferred only partial ANTXR2-like properties on the ANTXR1 receptor.

To test whether additional β4-α4 loop residues are required to convert ANTXR1 more fully into an ANTXR2-like receptor, ANTXR1 residues 154–159 were replaced with the corresponding amino acids of ANTXR2 (Fig. 2A). The ANTXR1 154–159 protein exhibited an additional 0.2 pH unit shift in the pH threshold of PA pore formation beyond that observed with ANTXR1 155–156 (Fig. 2B). Furthermore, in contrast to ANTXR1 155–156, PA pore formation associated with the ANTXR1 154–159 protein was sensitive to ammonium chloride inhibition (Fig. 2C). These data show that additional residues located in the β4-α4 loop of ANTXR2 can further convert ANTXR1 into a receptor, which acts more like ANTXR2.

To test the additional requirement for ANTXR2 residue Q88, this residue was substituted in the ANTXR1 154–159 protein, generating the ANTXR1 R88Q/154–159 protein. This additional change fully converted ANTXR1 154–159 into an ANTXR2-like receptor that restricted pore formation to pH 5.0 or below (Fig. 2B). These results provide an independent line of evidence that β4-α4 loop region residues including G153 and L154, as well as Q88, of ANTXR2 are largely responsible for dictating the 1.0 pH unit difference in the receptor-specific pH threshold of toxin pore formation.

### Structure-based mutagenesis of conserved receptor residues

**Figure 3:**
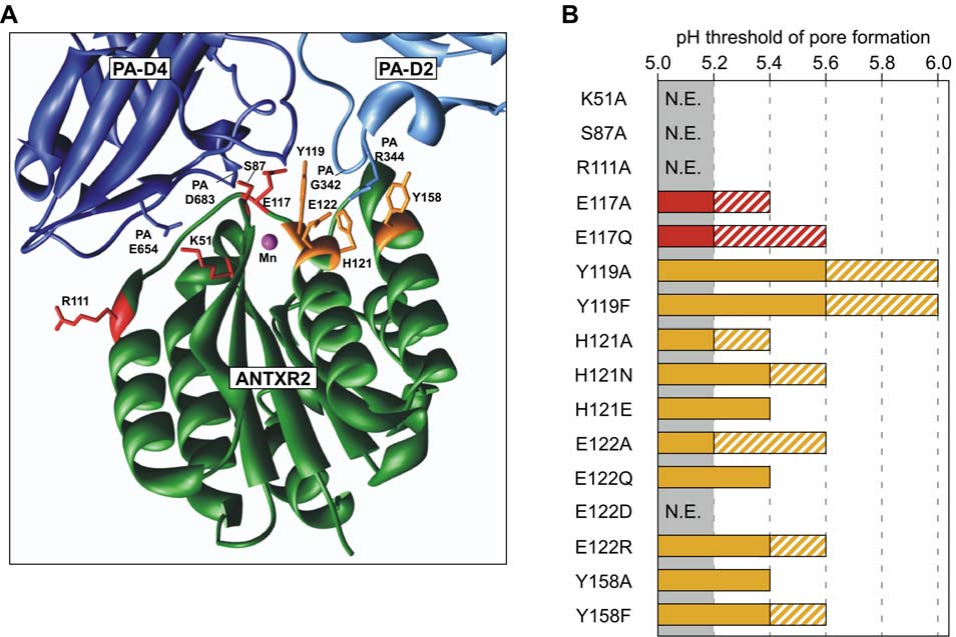
Mutagenesis of conserved receptor residues. (A) Ribbon model of the ANTXR2-PA interaction as described in the Fig. 1A legend. ANTXR2 residues that are conserved in ANTXR1 and are involved in PA-D2 and PA-D4 contact are depicted in orange and red stick representation, respectively. The PA residues E654 (dark blue) and R344 (light blue) that form salt-bridges with receptor residues K51 and E122, respectively, are also shown in stick representation. The position on the polypeptide backbone of PA residues G342 and D683 is also shown. (B) PA pore formation was assayed at different pH values on the surfaces of cells transiently expressing ANTXR2-EGFP and mutant ANTXR2-EGFP receptors, as described in Fig. 1B. The shaded region of the bar indicates the pH range where complete pore formation was observed, while the striped region of the bar indicates partial pore formation (as seen at pH 5.4 for Q88R and pH 5.6 for G153E in Fig. 1B). The results shown represent pooled data obtained from at least 3 independent experiments except in those cases where no effect (N.E.) was observed. In those cases, the data were collected from at least two independent experiments. PA bound to wild-type ANTXR2 formed pores between 5.0–5.2 (grey shaded area); receptors with mutations in this same pH range were determined to have no effect (N.E.).

At the PA binding interface, ANTXR2 amino acid residues K51, Y119, H121, E122 and Y158 are absolutely conserved with ANTXR1, and residues S87, R111 and E117 are residues are highly similar (Thr, Lys and Asp, respectively, in ANTXR1) [17], [18] (Fig. 3A). We reasoned that some of these residues are likely to constitute a conserved core of the molecular clamp mechanism that is shared by both anthrax toxin receptors. To test their involvement in the context of ANTXR2, each of these residues was independently changed to an Ala, and in some cases also to other amino acid side chains.

Among the PA domain 2-contact residues, the most important was Y119: changing this residue either to an Ala or Phe significantly weakened the molecular clamp allowing some toxin pore formation at pH 5.6–6.0 (Fig. 3B). Also, mutant receptors bearing either H121A, H121N, H121E, E122Q, E122R, Y158A, or Y158F amino acid substitutions displayed a 0.2–0.4 pH unit shift in this threshold (Fig. 3B). Among the PA domain 4-contact residues, changing residue E117, either to an Ala, or to the structurally-related Gln side chain, led to a weaker molecular clamp that allowed PA pore formation at pH 5.2–5.4 (Fig. 3B). Taken together these results indicate that the conserved residues E117, Y119, H121, E122, and Y158, which contact PA domain 2 and the neighboring edge of domain 4, influence the low pH threshold of ANTXR2-associated anthrax toxin pore formation.

## Discussion

In this report, receptor determinants that influence the strength of the ANTXR2 clamp which acts to restrict PA pore formation at neutral pH have been defined. Residues G153 and L154, located on the β4-α4 loop of the receptor I domain which interacts with the flexible 340–380 loop of PA domain 2 [17], [18] were shown to be the major determinants underlying the more acidic pH requirement for PA pore formation when the toxin is bound to ANTXR2. Substituting both of these residues in ANTXR2 for the corresponding ANTXR1 amino acids gave rise to a much weaker molecular clamp with the pH threshold properties of ANTXR1. Moreover, the ANTXR2 residues G153 and L154 strengthened the ANTXR1 molecular clamp when they were introduced together, especially in combination with residue Q88 and additional β4-α4 loop residues (ANTXR2 residues 152–157), at corresponding positions of the ANTXR1 receptor.

Residue G153 permits a tight turn in the β4-α4 loop and L154 participates in hydrophobic contacts with the side-chains of PA residues L340 and T349 (Fig. 2A). The sequence downstream of the β4-α4 loop of ANTXR2 contains a Pro residue (P155) that induces a backbone kink and is aliphatic and solvent-exposed, along with a neighboring Ser residue (S156). However, the corresponding residues of ANTXR1 are very hydrophobic (F158 and F159, respectively) (Fig. 2A). Thus, it seems likely that this region of ANTXR1 will adopt a very different conformation from that of ANTXR2, one that affects the surface presentation of PA domain 2-contact residues, potentially weakening the toxin interaction. Indeed, the contacts between the ANTXR1 I domain and PA domain 2 have yet to be characterized structurally.

**Figure 4:**
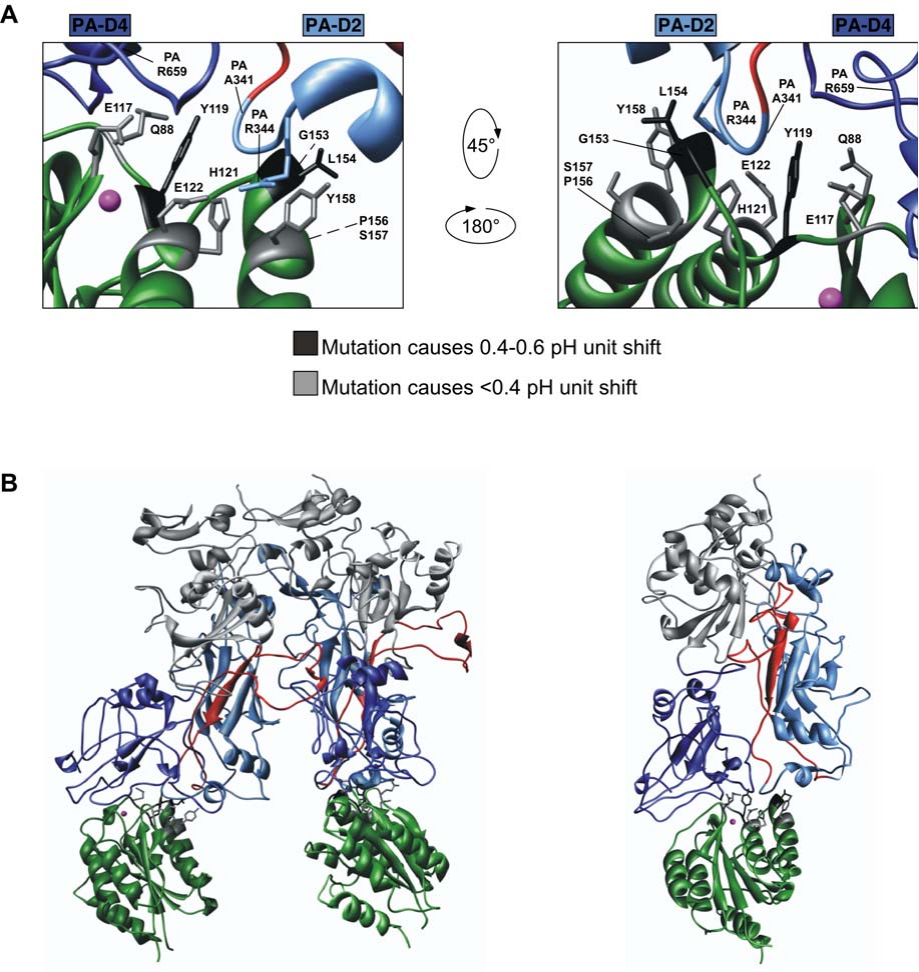
Receptor residues involved in molecular clamp function. (A) Structural diagram of the contribution of individual residues to the receptor molecular clamp (from the monomeric ANTXR2-PA structure solved at 2.5 Å resolution; PDB# IT6B)[17]. Molecular clamp residues from panel A are colored black if mutation of the residue caused a 0.4–0.6 pH unit shift in pore formation, or grey if the mutation caused a<0.4 pH unit shift in pore formation. (B) *Left,* Two ANTXR2 I domains bound to a PA_63_ dimer from the heptameric PA-ANTXR2 structure (solved at 4.3 Å resolution; PBD #ITZN)[18]. PA domains 2 and 4 are depicted in light and dark blue respectively, with the membrane insertion loop (residues 285–340) from PA domain 2 colored red. *Right,* One ANTXR2 I domain-PA monomer complex. Structural diagram of the contribution of individual residues to the receptor molecular clamp. Molecular clamp residues from panel A are colored black if mutation of the residue caused a 0.4–0.6 pH unit shift in pore formation, or grey if the mutation caused a<0.4 pH unit shift in pore formation.

Among the conserved receptor residues, the key regulator of acid pH-dependent pore formation was Y119 of ANTXR2, which occupies a planar position between domains 2 and 4 of PA (Fig. 4A) and appears to make hydrophobic and H-bond interactions with both domains. In particular, the hydroxyl moiety of this residue is critical since replacement by Phe also gave rise to a dramatic 0.6–0.8 unit shift in the pH threshold of toxin pore formation (Fig. 4A). It is not known how this hydroxyl moiety is involved, but it may make stabilizing H-bonds with the backbone of PA residue A341 and/or the side-chain of PA residue R659 in the PA prepore-receptor complex (Fig. 4A). Leppla and collaborators have also recently shown that the ANTXR2 Y119H mutation has a major effect upon the pH threshold of PA pore formation [27].

Substituting other conserved residues E117, H121, E122, and Y158 each gave rise to similar 0.2–0.4 unit changes in the pH threshold of toxin pore formation (Fig. 4A). E117 is H-bonded to the PA D683 backbone (Fig. 3A). The imidazole ring of H121 does not make any H-bonds with PA in the co-crystal structure, but this residue is in close proximity to ANTXR2 E122, the PA G342 backbone and PA R344 side-chain (Fig. 3A). A salt bridge between ANTXR2 residue E122 and PA residue R344 was recently implicated in strengthening the ANTXR2 receptor clamp [27]. The hydroxyl moiety of ANTXR2 Y158 appears to be important since a Phe substitution at this position led to a modest decrease in clamp strength. However, it is unknown how this hydroxyl participates because Y158 does not make H-bonds with the monomeric or prepore forms of PA [17], [18].

It remains to be determined if the residues implicated in this study affect the affinity of PA-ANTXR2 binding at neutral pH; the long half-life of the complex (15 hours) [19] precluded the use of moderate throughput analysis of all the mutants. Indeed, even SPR analysis is not sensitive enough to accurately measure the apparent K_d_ of the toxin-wild-type receptor complex and a more sensitive FRET-based assay involving the use of AF488 (donor fluorophore)-labeled PA and AF546 (acceptor fluorophore)-labeled ANTXR2 I domain had to be used in that study. Consequently, previous measurements obtained by binding PA to cell surfaces for 1 hour at 4°C [27], [28] likely reflect only association rate differences between different mutant ANTXR2 proteins. Since these experiments are not performed under equilibrium conditions, they also likely underestimate the actual PA-binding affinities of wild-type and mutant forms of ANTXR2.

The ANTXR2 residues that form the functional core of the receptor clamp, as well as those that dictate the receptor-specific pH thresholds of pore formation, map to the region that contacts PA domain 2 and the neighboring edge of PA domain 4 (Fig. 4A). These data therefore provide genetic evidence that receptor release of these two PA regions is likely necessary to allow the unfurling of the 285–340 region of PA domain 2 involved in pore formation (Fig. 4B). Our results also illuminate the genetic basis for the previous observation that there was a one unit difference in the pH threshold at which ANTXR2 and ANTXR1 allowed toxin pore formation, and support a model where toxin pore formation may be occurring in different endosomal compartments depending on the receptor bound [14]. Previously it was noted that the pH profile of anthrax toxin pore formation was consistent with the titration of histidines and that there are several histidines (H299, H304, H310, and H336), located in the 285–340 region of PA which forms the membrane-spanning pore [17], [18]. Moreover, it was suggested that ANTXR2 residue H121 might be a key component of this triggering mechanism since this residue is located at the PA domain 2 binding interface and is conserved in ANTXR1 [17], [18]. However, the current study excludes a major role for ANTXR2 residue H121 since its substitution by other amino acids had only a modest effect on the pH of pore formation. The precise molecular changes in PA-receptor complexes, which accompany anthrax toxin pore formation in response to a low pH stimulus, remain to be determined and are under investigation.

## Supporting Information

Table S1 Mutagenesis Primers
(0.07 MB DOC)
