# Supplementary material for: Anthrax Toxin Receptor 2 Determinants that Dictate the pH Threshold of Toxin Pore Formation
Source: PLoS One. 2007 Mar 28;2(3):e329. doi: 10.1371/journal.pone.0000329 (PMC1824706; doi:10.1371/journal.pone.0000329)
Supplement: Table S1 — Mutagenesis Primers (0.07 MB DOC) [file pone.0000329.s001.doc]

| **Table S1. Mutagenesis primers** | |  |
| --- | --- | --- |
| Template | Mutation | Forward Primer |
|  |  |  |
| ANTXR2-EGFP | K51A | 5’ CTTCGTCCTGGACGCGTCTGGGAGTGTG 3’ |
|  | S87A | 5’ CATTGTGTTTTCTGCTCAAGCAACTAT 3’ |
|  | R111A | 5’ GAGGATTTAAAAGCTGTTAGTCCAG 3’ |
|  | E117A | 5’ GTCCAGTAGGAGCGACATATATCCATG 3’ |
|  | E117Q | 5’ GTCCAGTAGGACAGACATATATCCATG 3’ |
|  | Y119A | 5’ CAGTAGGAGAGACAGCGATCCATGAAGGAC 3’ |
|  | Y119F | 5’ GTAGGAGAGACATTTATCCATGAAGG 3’ |
|  | H121A | 5’ GAGACATATATCGCGGAAGGACTAAAGCTAG 3’ |
|  | H121N | 5’ GAGACATATATCAACGAAGGACTAAAGCTAG 3’ |
|  | H121E | 5’ GAGACATATATCGAAGAAGGACTAAAG 3’ |
|  | E122A | 5’ GACATATATCCATGCGGGACTAAAGCTAGC 3’ |
|  | E122D | 5’ CATATATCCATGATGGACTAAAGCTAG 3’ |
|  | E122Q | 5’ GACATATATCCATCAAGGACTAAAGCTAG 3’ |
|  | E122R | 5’ GACATATATCCATCGCGGACTAAAGCTAGCG 3’ |
|  | Y158A | 5’ CTGGTGCCATCAGCGGCAGAGAAAGAGG 3’ |
|  | Y158F | 5’ CTGGTGCCATCATTTGCAGAGAAAGAG 3’ |
|  | A56L | 5’ GTCTGGGAGTGTGCTAAATAACTGGATTG 3’ |
|  | N57H | 5’ GGGAGTGTGGCACATAACTGGATTG 3’ |
|  | Q88R | 5’ GTGTTTTCTTCTCGAGCAACTATTATTTTG 3’ |
|  | S113L | 5’ GATTTAAAACGTGTTCTTCCAGTAGGAG 3’ |
|  | V115G | 5’ CGTGTTAGTCCAGGAGGAGAGACATATATC 3’ |
|  | D152H | 5’ GATGGCAAGTTGCACGGTCTGGTGCCATC 3’ |
|  | G153E | 5’ GGCAAGTTGGACGAGCTGGTGCCATC 3’ |
|  | L154D | 5’ CAAGTTGGACGGTGACGTGCCATCATATG 3’ |
|  | GL 153-154 ED | 5’ GGCAAGTTGGACGAGGACGTGCCATCATATG 3’ |
| ANTXR1-EGFP | ED 155-156 GL | 5’ GATGGAGAACTCCATGGACTTCTCTTTTTCTATTCAG 3’ |
|  | HED 154-156 DGL | 5’ GACTGATGGAGAACTCGATGGACTTCTCTTTTTCTATTCAG 3’ |
| ANTXR1^154-156^-EGFP | HEDLFF 154-159 DGLVPS | 5’ GGAGAACTCGATGGACTTGTCCCTTCCTATTCAGAGAGGGAGGC 3’ |
| ANTXR1^154-159^-EGFP | R88Q, 154-159 | 5’ GTTTTCTCCACCCAAGGAACAACCTTAATG 3’ |
|  |  |  |
